# 3′ RNA-seq is superior to standard RNA-seq in cases of sparse data but inferior at identifying toxicity pathways in a model organism

**DOI:** 10.3389/fbinf.2023.1234218

**Published:** 2023-07-27

**Authors:** Ryan S. McClure, Yvonne Rericha, Katrina M. Waters, Robyn L. Tanguay

**Affiliations:** ^1^ Biological Sciences Division, Pacific Northwest Laboratory, Richland, WA, United States; ^2^ Department of Environmental and Molecular Toxicology, Oregon State University, Corvallis, OR, United States

**Keywords:** 3′ RNA-seq, transcriptomics, toxicity pathways, zebrafish, functional enrichment

## Abstract

**Introduction:** The application of RNA-sequencing has led to numerous breakthroughs related to investigating gene expression levels in complex biological systems. Among these are knowledge of how organisms, such as the vertebrate model organism zebrafish (*Danio rerio*), respond to toxicant exposure. Recently, the development of 3′ RNA-seq has allowed for the determination of gene expression levels with a fraction of the required reads compared to standard RNA-seq. While 3′ RNA-seq has many advantages, a comparison to standard RNA-seq has not been performed in the context of whole organism toxicity and sparse data.

**Methods and results:** Here, we examined samples from zebrafish exposed to perfluorobutane sulfonamide (FBSA) with either 3′ or standard RNA-seq to determine the advantages of each with regards to the identification of functionally enriched pathways. We found that 3′ and standard RNA-seq showed specific advantages when focusing on annotated or unannotated regions of the genome. We also found that standard RNA-seq identified more differentially expressed genes (DEGs), but that this advantage disappeared under conditions of sparse data. We also found that standard RNA-seq had a significant advantage in identifying functionally enriched pathways via analysis of DEG lists but that this advantage was minimal when identifying pathways via gene set enrichment analysis of all genes.

**Conclusions:** These results show that each approach has experimental conditions where they may be advantageous. Our observations can help guide others in the choice of 3′ RNA-seq vs standard RNA sequencing to query gene expression levels in a range of biological systems.

## 1 Introduction

Over the last decade, the emergence of RNA sequencing has revolutionized our ability to determine the response of complex biological systems to changing environments through analysis of gene expression levels (Bourdon-Lacombe et al., 2015; Joseph, 2017). Systems where RNA-seq has been applied include clonal cell populations in monoculture (Landry et al., 2013), complex microbiomes (Carvalhais et al., 2012; Bashiardes et al., 2016), 3D human tissue models (Chang et al., 2021), and whole organisms including zebrafish (*Danio rerio*), a model vertebrate organism (Hu et al., 2019). One specific area where RNA-seq has been used is the response of zebrafish to a variety of specific chemicals or other kinds of stresses (Zheng et al., 2018; Shankar et al., 2019; Huang et al., 2020; Dasgupta et al., 2022). Application of RNA-seq has become a common -omics tool and recently more advanced technologies have been developed and applied to zebrafish and other systems. These include modifications of RNA sequencing designed to answer specific questions such as Prime-Seq, Decode-Seq, Lasy-Seq (Kamitani et al., 2019; Li et al., 2020; Janjic et al., 2022) single-cell RNA-seq, where specific transcriptomics signatures can be determined from sub-populations of cells (Bageritz and Raddi, 2019; Jiang et al., 2021; Metikala et al., 2021; Tatarakis et al., 2021), sequencing of specific RNA types such as microRNAs (Zayed et al., 2019) and combination of RNA-seq data sets to carry out metanalyses or to determine how genes are co-expressed across conditions (Shankar et al., 2019; De Toma et al., 2021; Shankar et al., 2021).

While RNA-seq has become a powerful tool to view gene expression levels, the process is still expensive, meaning that large numbers of samples needed for more integrated approaches or more specific comprehensive views of systems are still difficult to obtain. In standard RNA sequencing approaches, RNA is collected from a sample and is sheared to produce short oligomers of RNA, which are then sequenced and aligned back to the genome, resulting in reads spanning the entire length of a gene (Wang et al., 2009). Owing to the differing lengths of genes, certain genes may have only tens of reads aligned to them to determine their expression while other, longer, genes may have several thousand, even if both genes are expressed at relatively the same level. This means it is likely possible to reduce the total number of reads applied to a system with a more targeted approach so that both long and short genes expressed at the same level have a similar number of reads assigned to them. In response to this, RNA sequencing methods targeting the 3′ end of the gene (3′ RNA-seq), similar to how microarrays function (Fasold and Binder, 2012), have been developed. In these approaches only the 3′ end of the RNA transcript is sequenced as a proxy for expression of the entire gene (Torres et al., 2008). This approach results in a need for fewer reads to quantify the expression of a gene. Thus, 3′ RNA-seq is ideal for multiplexing of sequencing libraries (to reduce costs and allow for more data collection) or in instances where complex communities are under examination and low abundance members need to be sequenced with greater depth.

As 3′ RNA-seq is a relatively new technology that is fundamentally different compared to standard RNA sequencing, several studies have focused on a direct comparison of the two methods. Specifically, studies have compared the identification of differentially expressed genes (DEGs), alignment to a genome, or *de novo* transcriptome assembly, and how these factors may change as a function of gene length and number of reads collected. An early study by Moll, et al. showed that 3′ RNA-seq was superior to standard RNA-seq in detecting differentially abundant transcripts using a synthetic spiked-in RNA standard. However, this advantage of 3′ RNA-seq was only found when the number of reads in the standard approach was artificially reduced to match those found in 3′ RNA-seq (Moll et al., 2014). A later study by Tandonnet, et al. found that both standard and 3′ RNA-seq identified roughly the same number of DEGs, but standard RNA-seq aligned slightly better with qPCR confirmation and was superior in instances where a genome was not available and *de novo* assembly of reads was required (Tandonnet and Torres, 2017). Another later study by Ma et al. (2019) found that 3′ RNA-seq was better at detecting shorter transcripts, but standard RNA-seq was better at detecting DEGs, with no real difference between the two when compared to qPCR. A more recent study by Jarvis et al. (2020) found that both approaches showed a moderate overlap in DEGs but that 3′ RNA-seq was better at detecting DEGs when reads counts were lower. When moving beyond DEGs to enriched functions, this study reported moderate overlap between the two methods and that standard RNA-seq was superior at producing enriched functions compared to 3′ RNA-seq. Again, similar to the Ma, et al. study, the Jarvis et al. study also concluded that transcript length affected the detection of transcripts by standard *vs*. 3′ RNA-seq.

3′ RNA-seq offers a potential major advantage over standard RNA-seq in that it can often provide critical information on gene expression levels with fewer input reads. However, when to use either 3′ or standard RNA-seq to gain the best advantages of each to answer biological questions, particularly in whole animal, vertebrate systems is still being examined. Here, we applied each of these sequencing approaches to samples collected from whole zebrafish larvae exposed to a control condition or elevated perfluorobutane sulfonamide (FBSA). We set out to determine, for each method, 3′ or standard RNA-seq, how well alignment took place to the genome, the overlap of DEGs detected in both approaches, how this held up under conditions of sparse data and how many enriched functions were detected, which could be interpreted as the response of this organism to the toxic effects of FBSA. The current study differs from previous analyses in that we are more focused on the quality of enriched functions detected by the two methods, not merely the number and overlap of differentially expressed genes, as well as the application of each method when examining sparse data and the use of complex multicellular model organisms for chemical hazard assessment.

## 2 Materials and methods

### 2.1 Zebrafish husbandry

All experimental details pertaining to zebrafish husbandry, chemical exposures, and RNA collection have been previously published (Rericha et al., 2022). Briefly, tropical 5D wildtype zebrafish (*Danio rerio*) were maintained following protocols approved by Oregon State University’s Institutional Animal Care and Use Committee (IACUC-2021-0166) at the Sinnhuber Aquatic Research Laboratory (Corvallis, OR, United States). Adult zebrafish were maintained on a light:dark cycle of 14:10 h and at densities of 400 fish per 50-gallon tank on a recirculating filtered water system supplemented with Instant Ocean salts (Spectrum Brands, Blacksburg, VA, United States) (Barton et al., 2016). GEMMA Micro 300 (Skretting, Inc.; Fontaine Les Vervins, France) was fed to adult fish twice per day. Placement of spawning funnels in the tanks at night stimulated spawning the following morning when the lights turned on. Larvae were collected, staged (Kimmel et al., 1995), and kept in embryo medium (EM) containing 15 mM NaCl, 0.5 mM KCl, 1 mM MgSO_4_, 0.15 mM KH_2_PO_4_, and 0.7 mM NaHCO_3_ at 28°C (Westerfield, 2000).

### 2.2 Toxicant treatment

Perfluorobutane sulfonamide (FBSA) was obtained from SynQuest Laboratories (CAS: 30334-69-1, Lot: 358300, purity: 97%; Alachua, FL, United States). As previously published, a 22 mM stock solution was prepared in 100% methanol (LC/MS grade, CAS: 67-56-1), shaken on an orbital shaker overnight, and analytically measured using high-performance liquid chromatography and triple quadrupole mass spectrometry (LC-MS/MS) (Rericha et al., 2022).

At 4 h post fertilization (hpf), larvae were dechorionated enzymatically with pronase and an automated dechorionator (Mandrell et al., 2012). Larvae were then placed into round bottom 96-well plates (Falcon^®^, product number: 353227; Glendale, AZ, United States), 100 μL EM and a single larvae in each well, using an automated placement system (Mandrell et al., 2012). At 8 hpf, larvae were exposed to either solvent control conditions or 47 µM FBSA (all normalized to 0.5% methanol) through the removal of 50 μL EM and the addition of 50 µL appropriately diluted working stock solutions (1% methanol) to each well. Exposure to FBSA at 47 µM was previously shown to induce 80% incidence of any cumulative morphological effect at 120 hpf, which enabled phenotypic anchoring of transcriptomics. 96-well plates were then sealed (VWR, cat number: 89134-428; Radnor, PA, United States), shaken on an orbital shaker overnight at 235 rpm, and maintained in the dark at 28°C until further processing.

### 2.3 RNA collection

At 48 hpf, prior to the onset of morphological effects that were confirmed to occur at 120 hpf, mRNA was collected from a subset from both experimental conditions. Ten zebrafish larvae were pooled per replicate (n = 3) into 1.5 mL safe-lock tubes and euthanized on ice (Eppendorf; Enfield, CT, United States). To homogenize the samples, excess water was immediately removed, 200 µL RNAzol (Molecular Research Center; Cincinnati, OH, United States) and 100 µL 0.5 mm zirconium oxide beads were added. Homogenization was performed with a Bullet Blender (Next Advance, Averill Park, NY, United States) on setting 8 for 3 min. An additional 300 µL RNAzol was added to each tube prior to storage at −80°C.

Total RNA isolation was performed using a Direct-zol RNA MiniPrep kit (Zymo, cat number: R2052; Irvine, CA, United States), and the optional DNase I digestion step was included. Following isolation, the RNA concentration was measured using a SynergyMix microplate reader and Gen5 Take3 module (BioTek Instruments, Inc.; Winooski, VT, United States) quality of the RNA samples was assessed with an Agilent Bioanalyzer 2100 (Santa Clara, CA, United States) by the Center for Quantitative Life Sciences (CQLS) at Oregon State University, and all RINs were greater than 9.

### 2.4 RNA sequencing

For standard RNA-seq, library preparation and RNA sequencing were performed by the Beijing Genomics Institute (BGI; ShenZhen, China). The DNBseq platform entailed the purification and fragmentation of mRNA using oligo (dT)-attached magnetic beads, cDNA synthesis and processing, followed by amplification and further purification. Library quality was confirmed an Agilent Bioanalyzer 2100. Amplified products were then circularized to form the final library, amplified and formed into DNA nanoballs, loaded into patterned array, and sequenced with the BGISEQ-500 (100 bp paired end). For 3′ RNA-seq, library preparation and RNA sequencing were conducted by the CQLS at Oregon State University. Library preparation was performed using the Lexogen QuantSeq 3′ kit for Illumina. The details for this kit can be found there but importantly this library preparation does contain a step where polyA-tailed RNA transcripts are amplified with oligo (dT) primers, so both 3′ and standard RNA-seq enrich for mRNAs. The final 3′ RNA-seq final library was then sequenced using a HiSeq (100 bp single end).

### 2.5 Data analysis

All fastq files were first examined with FastQC. Standard RNA-seq data had a slightly higher quality score (measured by Phred) compared to 3′ RNA-seq but both approaches gave high quality data with Phred scores above 22 (except in the case of a few nucleotide positions of one sample, Supplementary Figures S1, S2). We also found that no samples had adaptor sequence contamination, because of this, and the lack of low-quality nucleotides, trimming was not applied. Alignment of data from both standard and 3′ RNA-seq was performed using the STAR aligner (Dobin et al., 2013) with the following arguments:

STAR --runThreadN 10 --genomeDir./genomeLex --readFilesIn < file1>.fastq.gz --outFilterType BySJout --outFilterMultimapNmax 20 --alignSJoverhangMin 8 --alignSJDBoverhangMin 1 --outFilterMismatchNmax 999 --outFilterMismatchNoverLmax 0.6 --alignIntronMin 20 --alignIntronMax 1000000 --alignMatesGapMax 1000000 --outSAMattributes NH HI NM MD --outSAMtype SAM --outFileNamePrefix Lexogen_Control_1_LexPipeline2

Alignment took place against the Genome Reference Consortium Zebrafish Build 11 (GRCz11) (https://www.ncbi.nlm.nih.gov/assembly/GCF_000002035.6/ . After all files were converted to SAM format gene counts were obtained using HTSeq (Anders et al., 2015) with the following arguments

Python -m HTSeq.scripts.count -m intersection-nonempty -s yes -f sam -r pos $sams/<file1>.sam $ref > $out/<file1>.txt

**FIGURE 1:**
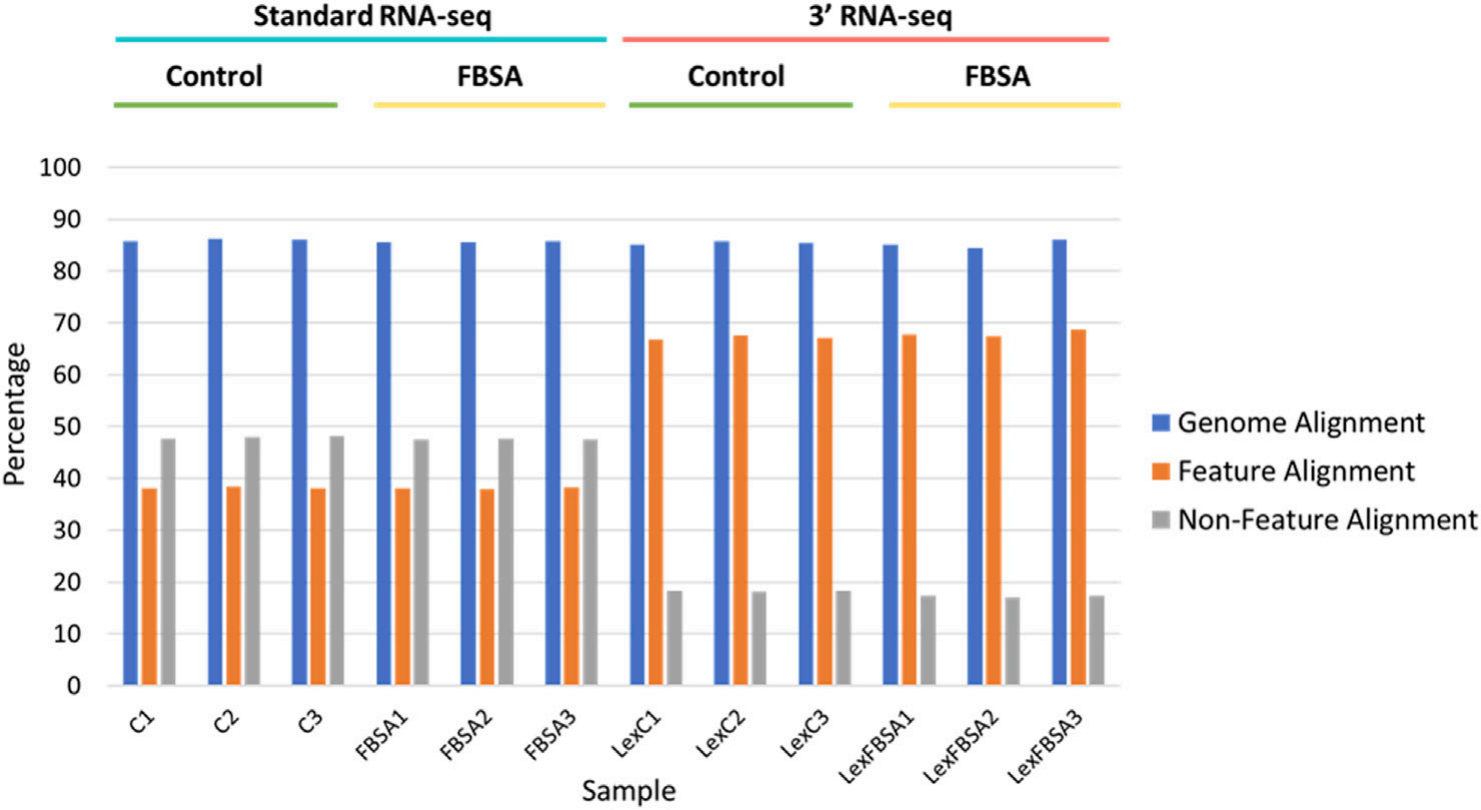
Alignment of standard and 3′ RNA-seq data. Percentage alignment is shown against the entire genome (blue bars), against regions of the genomes with annotated features (mRNA, tRNA, rRNA, other known non-coding RNA, orange bars) or against non-feature regions (grey bars). Individual samples are shown on the x-axis with sample groupings indicated above. C = standard RNA-seq samples from fish treated with control conditions, FBSA = standard RNA-seq samples from fish treated with FBSA, LexC = 3′ RNA-seq samples from fish treated with control conditions, LexFBSA = 3′ RNA-seq samples from fish treated with FBSA. Numbers at the end of the sample names indicate biological replicates.

Output files of HTSeq were used to determine alignment rates as well as alignment to features *vs*. non-features (Figure 1). Note that even though standard RNA-seq was carried out in a paired-end read manner (in contrast to 3′ RNA-seq which uses single-end reads) only the forward read was used in this analysis so that a better comparison of each approach could be made. Raw count files were then normalized using DESeq2 (Love et al., 2014) with default settings. DESeq2 was also used to identify differentially expressed genes (DEGs). DEGs were defined differently for particular analysis with the details in the Results. To generate files representing sparser data each raw count file was used (representing alignment to annotated regions of the genome). We first rarified the initial raw count dataset (six samples from standard RNA-seq and six from 3′ RNA-seq) to the same counts per sample (8,500,000) using the R function *rarefy*. The data frame of rarified gene counts was then rarified again so that raw count files of 90%, 80%, 70%, 60%, 50%, 40%, 30%, 20%, 10%, 5%, and 1% of that initial 8,500,000 reads were generated. These modified raw count files were then analyzed by DESeq2 as above. Functional enrichment of DEG lists identified by each method (standard and 3′ RNA-seq) was carried out using gProfiler (Raudvere et al., 2019). Output from gProfiler also included *q*-values, *p*-values adjusted for multiple hypothesis testing. Gene Set Enrichment Analysis (GSEA) algorithm was performed by averaging the log2 fold changes across the 3 replicates for each condition and using these values as input scores to GSEA, which was performed using the fgsea package (Korotkevich et al., 2016). The GO BP and MF datasets (http://geneontology.org/), as well as KEGG terms (https://www.genome.jp/kegg/) were used as input gene sets.

## 3 Results

3′ and standard RNA-seq show nearly identical genome alignment percentages but differing alignment to annotated or non-annotated regions.

Our initial viewing of the data showed that the samples from 3′ RNA-seq had a greater number of reads compared to standard RNA-seq (Table 1). This is likely not a function of the specific molecular biology of each approach but rather differences in the machines and laboratories carrying out sequencing. Despite the nearly 50% increase in reads for 3′ RNA-seq datasets, the number of genes detected (defined as an average raw count of 10 in either Control or FBSA conditions) was actually slightly lower in 3′ RNA-seq datasets compared to standard RNA-seq. Standard RNA-seq identified 18,347 genes while 3′ RNA-seq identified 17,435. As a first analysis, we looked at how 3′ and standard RNA-seq data aligned to the zebrafish genome. Since the 3′ RNA-seq methodology is focused on certain sections of the RNA transcript, we examined how successfully alignment to the complete genome occurred, as well as to annotated features of the genome (annotated genes) and non-feature regions of the genome (intergenic regions or other non-annotated areas). Regarding total alignment, both 3′ and standard RNA-seq showed almost identical results with approximately 85% of reads aligning to the genome (Figure 1). However, when aligning specifically to features (annotated genes) in the genome, 3′ RNA-seq showed a clear advantage with ∼67% of reads aligning compared to ∼38% for standard RNA-seq. When examining non-feature sections of the genome, this trend was reversed with standard RNA-seq showing ∼47% alignment compared to only ∼17% for 3′ RNA-seq. As 3′ RNA-seq specifically targets mRNA genes with 3′ poly-A tails this observation is not unexpected. It does suggest that additional, non-annotated RNA transcripts (possibly coding for unknown mRNAs) exist in the zebrafish genome. This would explain the disparity in 3′ RNA-seq between total alignment (85%) and alignment to annotated regions (67%), the remaining ∼18% may be aligning to unknown genes or at least transcripts with polyA tails. With either standard or 3′ RNA-seq, there was no difference in alignment in samples treated with FBSA or not. It should be noted that both methods (3′ and standard) included a step that enriched for mRNAs so that should not be a factor driving the difference in alignment to annotated genes. An analysis of the range of count values per gene between the two methods showed no major differences (Supplementary Figure S3).

**TABLE 1:** Count analysis of 3′ and standard RNA-seq.

	Standard RNA-seq						3′ RNA-seq					
	Control A	Control B	Control C	FBSA	FBSA	FBSA	Control A	Control B	Control C	FBSA	FBSA	FBSA
				A	B	C				A	B	C
Aligned to Annotated Genome Regions	32108756	24348679	23906021	33356411	29475787	27719471	10864586	10974427	8633918	10947502	10836291	10933700
Aligned to Unannotated Genome Regions	8812000	6533824	6542552	8505105	7448728	7013177	13602117	13696917	10907851	13606973	13571673	13588950
Total Aligned Counts	40920756	30882503	30448573	41861516	36924515	34732648	24466703	24671344	19541769	24554475	24407964	24522650
Alignment Ambiguous	119400	90495	89544	117259	106219	103877	37807	37142	28303	36003	36389	37193
Alignment not Unique	7065100	5047016	5099812	7240014	6667926	5490664	4048189	3893791	3122834	4104526	4090169	4050230
Total Counts	48105256	36020014	35637929	49218789	43698660	40327189	28552699	28602277	22692906	28695004	28534522	28610073

### 3.1 3′ RNA-seq and standard RNA-seq methods show moderate overlap on DEG identification

**FIGURE 2:**
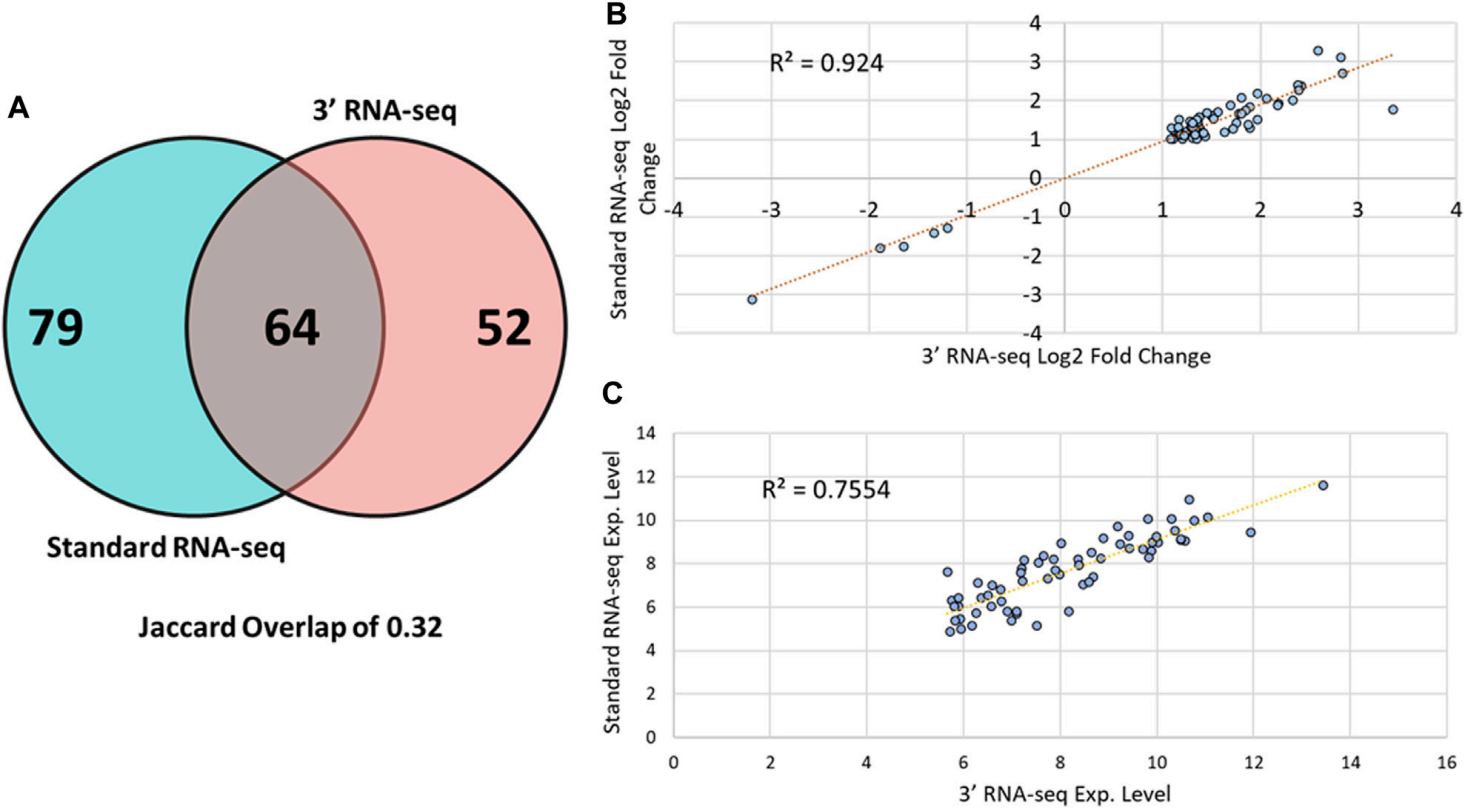
DEG overlap among 3′ and standard RNA-seq. **(A)** A Venn diagram showing overlap between DEGs identified by the standard RNA-seq approach (blue circle) and DEGs identified by 3′ RNA-seq (red circle). **(B)** Scatter plot showing the log2 fold change of all 64 DEGs identified by both 3′ and standard RNA-seq. **(C)** Scatter plot showing the log2 mean expression value of all 64 DEGs identified by both 3′ and standard RNA-seq.

We next examined whether 3′ and standard RNA-seq identified the same DEGs when comparing control sample to those from FBSA-treated fish (Figure 2A). Here, we defined a DEG as any gene showing at least an absolute log2 fold change value of > 1 in abundance with an adjusted *p*-value (*q*-value) of less than 0.05. There was Jaccard overlap of 0.32 when comparing standard to 3′ RNA-seq with regard to DEGs. Standard RNA-seq identified 143 DEGs and 3′ RNA-seq 116, with 64 DEGs in common between the two methods. We used these shared DEGs to examine sample-sample correlation and hierarchical clustering of samples (Supplementary Figures S4, S5). We found that both PCA and hierarchical clustering showed an effect of sequencing strategy, but this effect was smaller than that shown by FBSA treatment. Thus, at least in this case, biological effects of the experiment that are of interest outweigh effects of sequencing methodology. When examining genes that were detected as DEGs by standard but not by 3′ RNA-seq, the main difference was overall gene expression level. Only 5/79 genes detected as DEGs solely by standard had a log2 mean expression level less than 30 in the standard dataset. In contrast, among these same 79 genes in the 3′ dataset, 33/79 genes had a mean expression level less than 30. Most genes that were detected as DEGs in the standard but not the 3′ RNA-seq data also had higher q-values (>0.05) and lower log2 FC values (<1). For the 52 genes that were detected as DEGs in the 3′ dataset but not in the standard RNA-seq, results were similar but with some differences. In the standard dataset, approximately half of these 52 genes were statistically significantly different in their expression (q-value < 0.05) but their absolute log2 fold change values were below 1. Genes detected as DEGs in the 3′ dataset only were also expressed at lower levels in the standard dataset. Only 2/52 of these genes had an expression value below 50 in the 3′ RNA-seq dataset while 15/52 of these same genes had an expression value below 50 in the standard RNA-seq dataset. Expression and fold change data for all DEGs in both datasets is shown in Supplementary Table S1. While standard and 3′ RNA-seq showed moderate overlap of DEGs, there was very strong correlation in the magnitude and direction of fold change regarding the DEGs that were identified in common between the two methods with an R_2_ value of 0.94 (Figure 2B). However, the overlap in the mean expression value (how highly a gene was expressed overall) did show more variation between standard and 3′ RNA-seq with a R_2_ value of ∼0.75, suggesting that while the fold change between the two conditions may be constant the actual expression value of the gene (log 2 expression value after DESeq2 normalization) may be different in 3′ or standard RNA-seq. As mentioned above, on average, DEGs detected by 3′ RNA-seq showed higher overall expression compared to standard RNA-seq but this was not statistically significant.

### 3.2 3′ RNA-seq is superior to standard RNA-seq when examining sparse data

**FIGURE 3:**
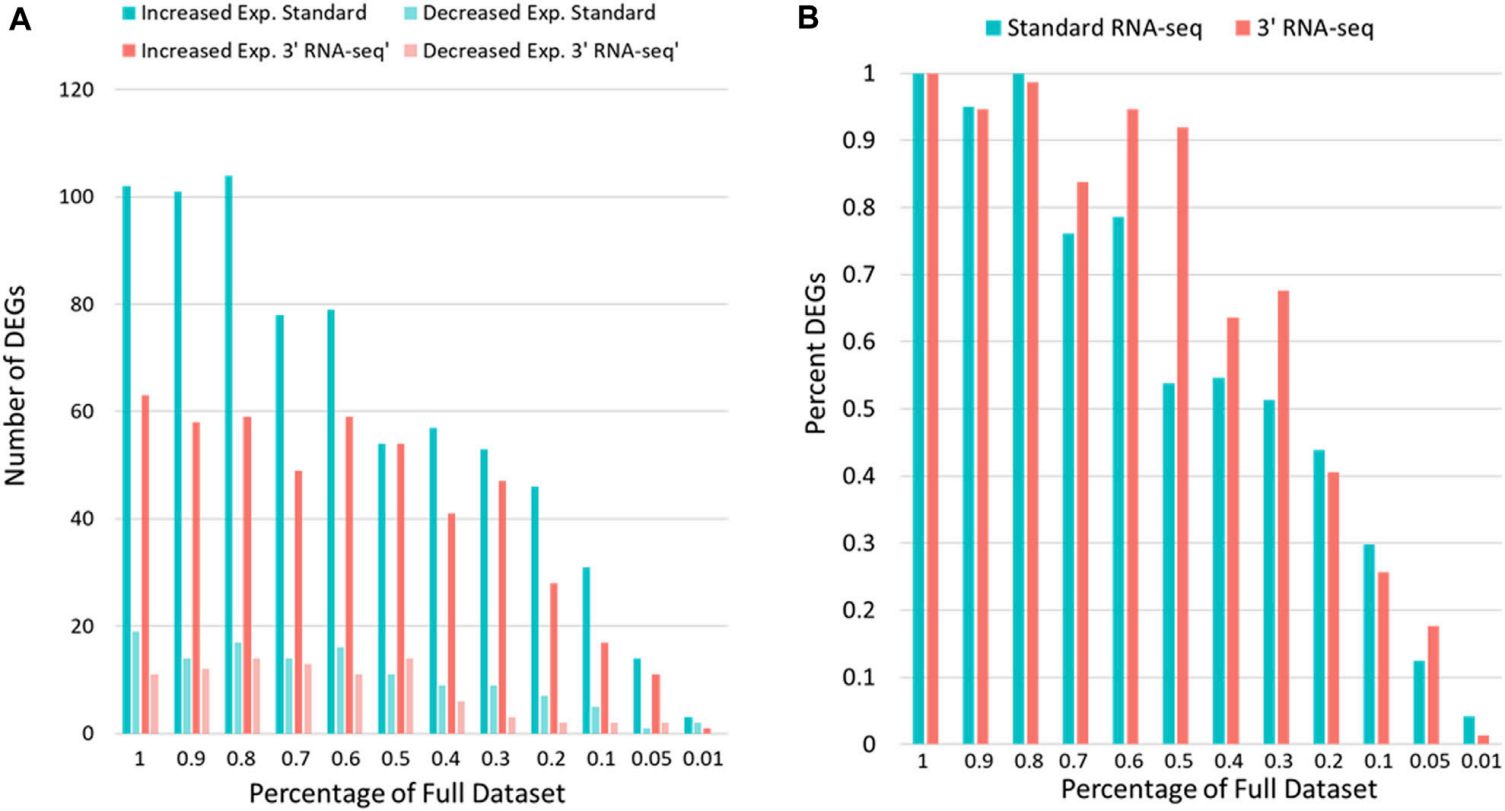
DEG identification with sparse data. **(A)** The number of genes showing statistically significant increased or decreased expression for standard and 3′ RNA-seq data. **(B)** The y-axis shows the DEGs identified as data is reduced as a percentage of the DEGs identified with the full dataset. The x-axis shows the percent of full data examined.

Because 3′ RNA-seq sequences only a small part of the transcript and extrapolates this data into an expression value for the entire gene, it is likely that fewer overall reads could be utilized by 3′ RNA-seq to gain the same conclusions compared to standard sequencing. We next compared how many DEGs were identified by 3′ *vs*. standard RNA-seq when the gene counts were iteratively reduced from 100% (the complete data) down to 1% of the original gene count levels (Figure 3). Since there was an overall difference in total counts for each sample set (Table 1), we first used rarefaction on raw count files so that samples from each method had exactly 8,500,000 counts. We then looked at the number of genes detected (defined above). When rarefaction was used to make the number of counts between the two datasets identical, the disparity in detected genes was even greater than before. Standard RNA-seq identified 17,763 genes while 3′ RNA-seq identified only 14,132. Similar to Figure 2, standard RNA-seq also consistently found more DEGs compared to 3′ RNA-seq (Figure 3A). However, as a percentage of DEGs found compared to the full dataset, 3′ RNA-seq emerged with the advantage as datasets were reduced in size (Figure 3B). With the data reduced by 50% (approximating a sample with half as many RNA-seq reads as our complete dataset), 3′ RNA-seq was still able to identify more than 90% of the original DEGs identified by this method in the full dataset. This is compared to slightly more than 50% of the DEGs identified in the full dataset for standard RNA-seq. Even when the data was reduced by 70%, 3′ RNA-seq was still able to identify nearly 70% of the original DEGs.

### 3.3 Standard RNA-seq is superior to 3′ RNA-seq when examining number of enriched functions from DEG lists

Often in transcriptomic analysis, after DEGs are identified, functional enrichment is carried out on DEG lists to highlight certain processes that may be activated or repressed. Enrichment analyses applied to chemical exposure are often used to identify dose- and time-dependent toxicity pathways and have been proposed as a method of translating transcriptomic changes after chemical exposure into information that can be used for hazard and risk assessment (Gao et al., 2015; Dean et al., 2017). We carried out separate functional enrichment analyses of the DEGs identified by 3′ RNA-seq and the DEGs identified by standard RNA-seq. This functional enrichment was applied to datasets that were rarified to the same level (8,500,000 reads) so that equivalent comparisons could be made. After this rarefaction, standard RNA-seq identified 121 DEGs and 3′ RNA-seq identified 74 DEGs. DEGs identified by 3′ RNA-seq were enriched for eight functions while DEGs identified by standard RNA-seq were enriched for 47 functions, including six of the same functions that were enriched in 3′ RNA-seq DEGs (Table 2). Despite having a similar number of DEGs and a fairly large overlap in DEGs identified by both methods, standard RNA sequencing identified far more functions compared to 3′ RNA sequencing. Of the functions that were identified by both methods, several of them were related to lysosomal processing or intracellular vacuoles.

**TABLE 2:** Functions identified in DEGs lists from standard and 3′ RNA-seq. Functions in green were identified via both approaches.

Source	Function	Adjusted *p*-value	Number of genes in function	Number of genes in input list	Number of genes common to function and input list
Functions enriched in standard RNA-seq DEGs					
GO:MF	Oxidative phosphorylation uncoupler activity	0.006	2	100	2
GO:MF	ATP-activated inward rectifier potassium channel activity	0.007	13	100	3
GO:BP	carboxylic acid metabolic process	0.001	561	91	13
GO:BP	oxoacid metabolic process	0.001	566	91	13
GO:BP	cellular amino acid metabolic process	0.001	248	91	9
GO:BP	organic acid metabolic process	0.001	599	91	13
GO:BP	cellular amino acid catabolic process	0.003	99	91	6
GO:BP	alpha-amino acid metabolic process	0.004	159	91	7
GO:BP	unsaturated fatty acid biosynthetic process	0.005	28	91	4
GO:BP	small molecule metabolic process	0.009	1222	91	17
GO:BP	unsaturated fatty acid metabolic process	0.016	37	91	4
GO:BP	adaptive thermogenesis	0.017	2	91	2
GO:BP	alpha-amino acid catabolic process	0.029	86	91	5
GO:BP	transmembrane transport	0.036	1501	91	18
GO:BP	carboxylic acid catabolic process	0.046	156	91	6
GO:BP	organic acid catabolic process	0.046	156	91	6
GO:CC	lysosome	0.000	156	81	7
GO:CC	lytic vacuole	0.000	157	81	7
GO:CC	extracellular region	0.004	1548	81	18
GO:CC	vacuole	0.004	222	81	7
GO:CC	extracellular space	0.005	1008	81	14
KEGG	Lysosome	0.006	145	31	6
KEGG	Arginine and proline metabolism	0.007	50	31	4
KEGG	Apoptosis	0.009	157	31	6
KEGG	Phagosome	0.036	136	31	5
REAC	Collagen degradation	0.000	42	32	5
REAC	Assembly of collagen fibrils and other multimeric structures	0.001	50	32	5
REAC	Collagen formation	0.004	75	32	5
REAC	RUNX1 regulates transcription of genes involved in differentiation of keratinocytes	0.011	16	32	3
REAC	Trafficking and processing of endosomal TLR	0.022	20	32	3
REAC	Physiological factors	0.049	5	32	2
HP	Increased serum prostaglandin E2	0.005	8	23	3
HP	Abnormal circulating prostaglandin circulation	0.005	8	23	3
HP	Renal juxtaglomerular cell hypertrophy/hyperplasia	0.005	8	23	3
HP	Low-to-normal blood pressure	0.008	9	23	3
HP	Abnormal urine chloride concentration	0.008	9	23	3
HP	Hyperchloriduria	0.008	9	23	3
HP	Hyperprostaglandinuria	0.011	10	23	3
HP	Abnormal circulating eicosanoid concentration	0.015	11	23	3
HP	Abnormal circulating unsaturated fatty acid concentration	0.015	11	23	3
HP	Hyperactive renin-angiotensin system	0.020	12	23	3
HP	Hypochloremia	0.020	12	23	3
HP	Hyposthenuria	0.026	13	23	3
HP	Abnormal urine osmolality	0.026	13	23	3
HP	Hypokalemic metabolic alkalosis	0.032	14	23	3
HP	Increased urinary potassium	0.032	14	23	3
HP	Hypokalemic alkalosis	0.050	16	23	3
					
Functions Enriched in 3′ RNA-seq DEGs					
GO:BP	negative regulation of developmental process	0.037	139	58	5
KEGG	Lysosome	0.008	145	22	5
REAC	Collagen degradation	0.000	42	26	5
REAC	Assembly of collagen fibrils and other multimeric structures	0.000	50	26	5
REAC	Collagen formation	0.001	75	26	5
REAC	RUNX1 regulates transcription of genes involved in differentiation of keratinocytes	0.006	16	26	3
REAC	Trafficking and processing of endosomal TLR	0.012	20	26	3
REAC	Degradation of the extracellular matrix	0.025	134	26	5

**FIGURE 4:**
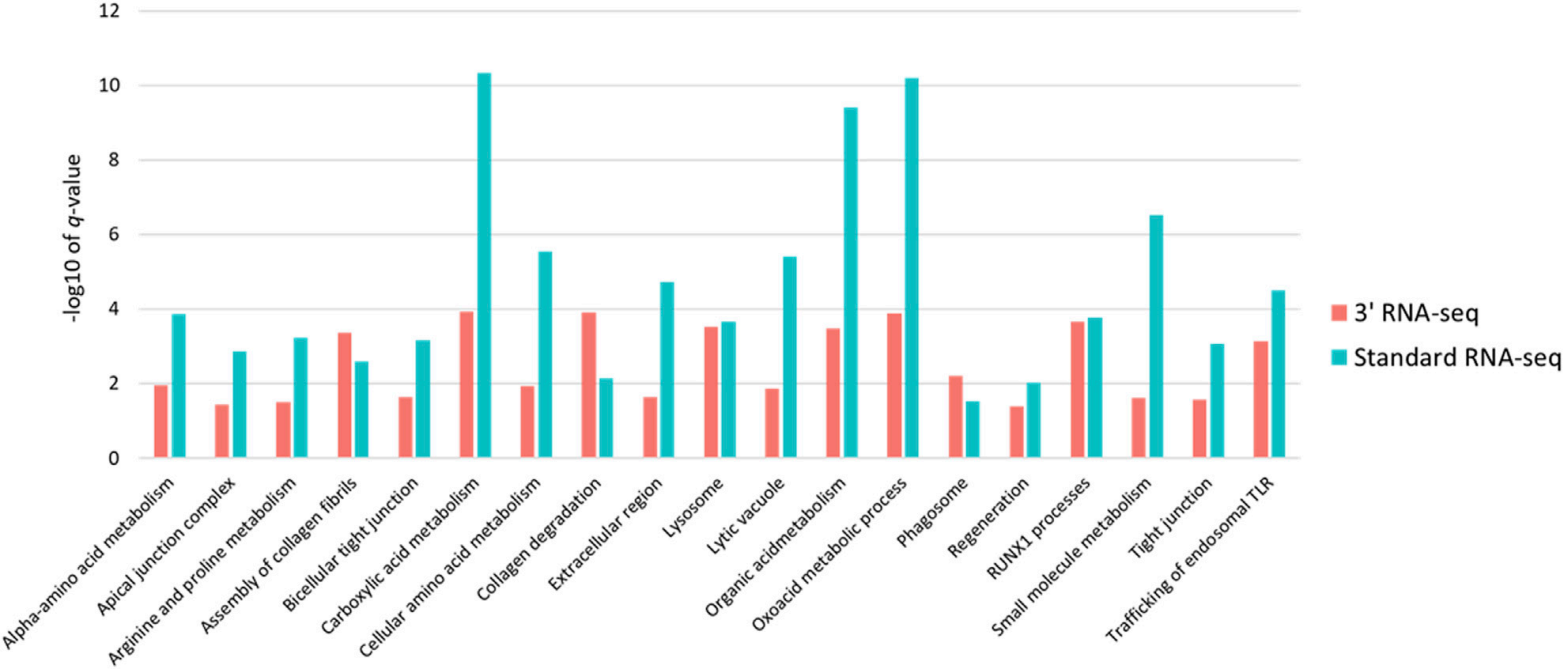
Statistical significance and intersection of 3′ and standard RNA-seq enriched functions. For all functions found by both standard and 3′ RNA-seq DEG functional enrichment the −log10 of the q-value is shown. Red bars represent q-values of functions enriched from the 3′ RNA-seq DEG list and blue bars represent functions enriched from the standard RNA-seq DEG list.

Because so many more functions were identified using standard RNA-seq, despite the fact that the number of DEGs identified by both methods was very similar, we examined in more detail the functional enrichment profiles of DEGs from both approaches. We first expanded the list of DEGs for both approaches by relaxing our definition of a DEG to any gene with a 1.5-fold change in abundance with an adjusted *p*-value of less than 0.1. Using this cutoff for a DEG, standard RNA-seq identified 62 functions and 3′ RNA-seq identified 27 functions, with 20 functions found by both methods. We then looked specifically at the -log10 of the *q*-value of the enrichment of each function found by both methods. Based on this metric, standard RNA-seq showed a superior outcome; the -log10 of the *q*-values was higher in functions identified by standard RNA-seq compared to the -log10 of the same functions identified by 3′ RNA-seq (Figure 4). On average, standard RNA-seq *q*-values showed a -log10 of 4.65 while 3′ RNA-seq values showed a value of 2.51. This difference was statistically significant with a *p*-value of less than 0.01. These data demonstrate that not only does a DEG list from standard RNA-seq identify more functions, but the functions it does identify are more statistically significant.

**FIGURE 5:**
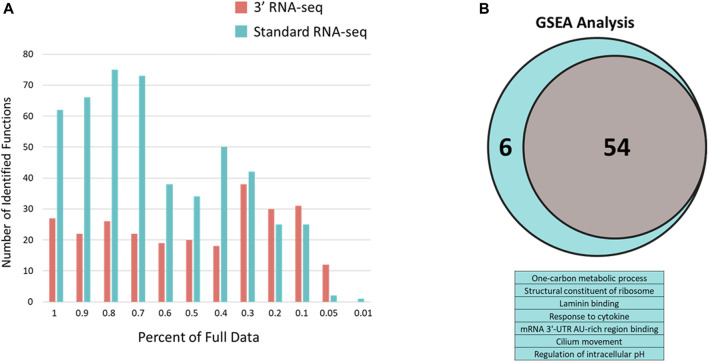
Number of enriched functions identified with 3′ and standard RNA-seq DEG lists using sparse data and q-value defined data. **(A)** Number of enriched functions using DEG lists from 3′ RNA-seq (red bars) and standard RNA-seq (blue bars) data across sparse data sets. Percent of the full data is shown in the x-axis. **(B)** GSEA analysis of full datasets from standard and 3′ RNA-seq. Venn diagram is shown for functions identified by both approaches (54) with the table below showing the six additional functions identified solely by standard RNA-seq.

To determine whether the large difference between standard and 3′ RNA-seq regarding identified functions was an effect of this particular pair of DEG lists, we took advantage of the sparse data sets we generated earlier (Figure 3). Here again, standard RNA-seq consistently identified more functions compared to 3′ RNA-seq until the percentage of data dropped more than 70% compared to the original dataset (Figure 5A). Since the definition of a DEG can be somewhat arbitrary (fold change or *q*-value cutoff, etc.) and 3′ RNA-seq and standard RNA-seq rarely identified the exact same number of DEGs, we also used Gene Set Enrichment Analysis (GSEA) with both approaches to examine how functions may be identified when there is no cutoff used to designate a DEG. With GSEA analysis the results between 3′ and standard RNA-seq were much more similar (Figure 5B). Using the complete dataset with no sparsity, there were 54 functions identified by GSEA in the 3′ RNA-seq dataset and 60 functions identified in the standard RNA-seq dataset (identification defined as being enriched with a *q*-value of less than 0.05). There was also strong overlap between the two approaches with all functions identified by 3′ RNA-seq also identified in the standard RNA-seq dataset as well as six additional functions. As in Figure 5A the significance of identified functions (average -log10 of the *q*-value) was higher in the Standard RNA-seq datasets (4.21) compared to the 3′ RNA-seq dataset (3.67) with the *p*-value of this comparison just slightly over significance at 0.11 using a Student’s t-test.

## 4 Discussion

As the use of RNA sequencing becomes more widespread, a number of tools and modifications to the fundamental technology have become available. However, determining where and under what conditions certain modifications should be used is paramount to proper experimental setup and data analysis. Here, we examined standard RNA sequencing (based on collecting all transcripts and using alignment to the whole gene as measure of expression) and 3′ RNA sequencing (based on enriching for 3′ ends of the transcript and using this as a proxy for expression of the whole gene) to determine under which experimental conditions each approach demonstrated advantages or disadvantages. Rather than definitively identifying one or the other of these approaches as superior, our results suggest that each technology should be applied specifically when certain experimental parameters are present. 3′ RNA seq is far better at alignment to annotated regions of the genome compared to standard RNA seq. We had the opportunity to study this aspect because zebrafish has a very well annotated genome (Kelkar et al., 2014; Lawson et al., 2020). However, in many cases, poorly annotated genomes must be contended with when carrying out (meta) transcriptomic studies. Previous studies have laid out best practices for carrying out transcriptomic analysis of genomes with poor annotation (Conesa et al., 2016). These included sequencing longer read lengths to improve alignment (Łabaj et al., 2011), using paired-end reads rather than single end reads (Katz et al., 2010) and avoiding a gapped mapper (Langmead and Salzberg, 2012). Our studies here indicate that one should also avoid using 3′ RNA-seq under conditions where a genome is not well annotated or where the genome does not exist, and *de novo* transcriptome assembly is needed. This advantage of standard RNA-seq against unannotated genomes was also found in another study that examined whole organism samples (Tandonnet and Torres, 2017).

We also found that 3′ RNA-seq is well suited to identifying DEGs under conditions of data sparsity. A low number of reads is a common issue in transcriptomics especially in the case of metatranscriptomic studies where many species are being examined simultaneously in a mixed community or in cases where costs mean that many samples must be multiplexed in the same sequencing run. In cases with microbiome studies, 50-100 million reads must be split across thousands of genomes. Many of these genomes will be covered at a level that allows for some determination of gene expression but not at levels that are ideal for robust analysis (Tveit et al., 2014; Nichols et al., 2019). Under sparse reads conditions, use of 3′ RNA-seq would be strongly recommended as it is able to identify a significant number of DEGs even when only a half of the original reads are present. Low reads could also emerge when RNA of low quality is collected (Adiconis et al., 2013). Furthermore, another study recently found that degraded RNA sequences actually have a 3′ mapping bias, further supporting the use of 3′ RNA-seq when faced with samples of degraded RNA (Sigurgeirsson et al., 2014). The observation that 3′ RNA-seq is superior to standard when faced with low reads was also found by a previous analysis directly comparing these two methods (Jarvis et al., 2020).

Standard *vs*. 3′ RNA-seq show some differences in alignment, the number of DEGs detected and how this may change when data becomes sparse with each approach having advantages in certain situations. However, standard RNA-seq was by far the superior method when examining the number of enriched functions identified from DEG lists as well as the statistical significance of the enrichment of these functions. Interestingly, this was not the case when, instead of DEG input lists, gene set enrichment analysis (GSEA) was used. GSEA does not rely on user defined cutoffs to generate DEG lists and instead uses global expression levels from all genes. This suggests that the same general set of pathways are detected as activated when either standard or 3′ RNA-seq is used, with part of the reason for the differences in functional enrichment lying in whether genes examined by 3′ or standard RNA-seq have reach user-defined thresholds of a differentially expressed gene (greater than 2-fold change in abundance or a q-value of less than 0.05). Standard RNA-seq generally finds a greater number of DEGs which could lead to a greater number of statistically-enriched functions. However, in our analysis of sparse data certain datasets led to standard and 3′ RNA-seq identifying virtually the same number of DEGs. In these cases, standard RNA-seq still identified more functions so the absolute number of DEGs identified is likely only part of the answer. Regardless of the outcomes of functional enrichment with gProfiler the results of GSEA show that both standard and RNA-seq identify activation of many of the same functions, though standard RNA-seq still does have a slight advantage in the number of functions detected and the statistical significance. We chose to include both functional enrichment with a set of DEGS and GSEA as both methods are still widely used to interpret RNA-sequencing data. However, it is known that relying on an arbitrary definition of a DEG (user defined measures based on fold change or q-value) can miss key genes and thus pathways. Other researchers have also pointed out this flaw and encouraged the use of GSEA, which does not rely on DEG definitions and instead uses expression data from all genes (Pan et al., 2005; Simillion et al., 2017). Our results here support this conclusion and suggest that GSEA should we used in place of functional enrichment with DEG lists wherever possible and certainly with 3′ RNA-seq data.

In addition to a greater number of functions identified by standard RNA-seq with DEG input lists, there was also stronger overlap between this study and a previous, independent analysis using the same RNA samples (Rericha et al., 2022). We compared these results to a study by our group examining the response of zebrafish to a number of chemicals including FBSA (Rericha et al., 2022). The focus of the previous paper was the response of zebrafish to toxicants while the focus of the current study was direct comparison of RNA-seq methods. Because of these varying goals, there were a number of differences in the analysis of the data, and the databases used to identity functions, between the study here and our previous one. When we compared a list of enriched gene ontology (GO) terms that were identified in the earlier study with the terms identified here, we found that standard RNA-seq actually had a slight advantage in common functions identified. Standard RNA-seq identified 61 functions (when DEGs are defined as any gene with a 1.5-fold change in abundance with an adjusted *q*-value of less than 0.1) and 3′ RNA-seq identified 26 functions. Two functions (∼3%) were found to be in common between the standard RNA-seq function list and the function list from our earlier study. In contrast, no functions were found to be in common between the 3′ RNA-seq list and our earlier study. Again, differences between the analyses lead to different functions being enriched. It should also be noted that in many cases very similar functions were found between this study and the earlier study but did not count towards an overlap due to not having the exact same GO term (e.g., fatty acid metabolic process *vs*. fatty acid biosynthetic process). We also found strong overlap in the actual DEGs identified by both 3′ and standard RNA-seq with the previous study (which as mentioned earlier used the same RNA samples we sequence here). For standard RNA-seq 424/547 of the DEGs identified (77%) were also identified as DEGs in the previous study, for 3′ RNA-seq this overlap was 189/252 genes (75%). For all analyses it should be noted that this is a single study comparison of two conditions. Conclusions here should not be taken as definitive answers of how 3′ or standard RNA-seq behaves generally across all experiments. Rather, these findings should be added to the growing list of analyses contrasting these two methods in other publications. By taking this approach we can address how reproducible the conclusions presented here are. We have found that 3′ RNA-seq has a distinct advantage in alignment when using annotated genomes and in working with sparse data. These aspects have also been found in additional studies mentioned above. The fact that these characteristics have been reproducibly found in several studies suggest that they should be given considerable weight when choosing RNA-seq methods in future experiments. In contrast, the enrichment advantages of standard RNA-seq found in this study have not yet been examined in detail in other systems. Therefore, that aspect of standard *vs*. 3′ RNA-seq should be taken as a preliminary conclusion generally until other studies confirm it.

## 5 Conclusion

As new RNA sequencing technologies emerge, the best way to use them will be in circumstances where they give the most advantage. Here, we directly compare standard and 3′ RNA-seq and find that 3′ RNA-seq is better with annotated genomes and with sparse data. In contrast, standard RNA-seq is better at finding functions using DEG lists and with more significant enrichment scores, which are important for the evaluation of dose- and time-dependent mechanisms of toxicity (Gao et al., 2015; Dean et al., 2017). This advantage is less so when using GSEA and when working with unannotated genomes. While we apply this method to the response of zebrafish to chemical exposure it should be emphasized that 3′ RNA-seq can and should be used to answer a number of different questions across biological systems. Proper use of 3′ RNA-seq will allow for much more efficient RNA sequencing applied to experiments, including those involving important model organisms such as zebrafish. More comprehensive RNA sequencing datasets will allow for more advanced approaches related to data integration and modeling efforts that can reveal new processes central to vertebrate organism response to xenobiotics and other potentially toxic chemicals.

## Data Availability

Publicly available datasets were analyzed in this study. This data can be found here: GEO: GSE186576 https://www.ncbi.nlm.nih.gov/geo/query/acc.cgi?acc=GSE186576.

## Supplementary material

SUPPLEMENTARY FIGURE S1 Phred scores of Standard RNA-seq samples.

SUPPLEMENTARY FIGURE S2 Phred scores of 3′ RNA-seq samples.

SUPPLEMENTARY FIGURE S3 Boxplot of count per gene. Samples are shown on the x-axis with log10 count values on the y-axis.

SUPPLEMENTARY FIGURE S4 Heatmap of samples built from shared DEGs. Samples are shown on the x-axis, “X3” indicates 3′ RNA-seq. Darker green indicates higher log2 expression (the “value” in the key). Samples are hierarchically clustered based on the dendrogram at the top.

SUPPLEMENTARY FIGURE S5 PCA of samples built from shared DEGs. Red dots and frame indicate Control samples from 3′ RNA-seq, green dots and frame indicate FBSA samples from 3′ RNA-seq, blue dots and frame indicate Control samples from Standard RNA-seq and purple dots and frame indicate FBSA samples from Standard RNA-seq.

SUPPLEMENTARY TABLE S1 Expression and fold change data of all genes and samples.

## References

[B1] Adiconis X. Borges-Rivera D. Satija R. DeLuca D. S. Busby M. A. Berlin A. M. (2013). Comparative analysis of RNA sequencing methods for degraded or low-input samples. Nat. methods 10 (7), 623–629. 10.1038/nmeth.2483 23685885 PMC3821180

[B2] Anders S. Pyl P. T. Huber W. (2015). HTSeq--a Python framework to work with high-throughput sequencing data. Bioinformatics 31 (2), 166–169. 10.1093/bioinformatics/btu638 25260700 PMC4287950

[B3] Bageritz J. Raddi G. (2019). Single-cell RNA sequencing with drop-seq. Methods Mol. Biol. 1979, 73–85. 10.1007/978-1-4939-9240-9_6 31028633

[B4] Barton C. L. Johnson E. W. Tanguay R. L. (2016). Facility design and health management program at the sinnhuber aquatic research laboratory. Zebrafish 13 (S1), S-39–S-43. S-39-S-43. 10.1089/zeb.2015.1232 26981844 PMC4931725

[B5] Bashiardes S. Zilberman-Schapira G. Elinav E. (2016). Use of metatranscriptomics in microbiome research. Bioinform Biol. Insights 10, BBI.S34610–25. 10.4137/bbi.s34610 PMC4839964 27127406

[B6] Bourdon-Lacombe J. A. Moffat I. D. Deveau M. Husain M. Auerbach S. Krewski D. (2015). Technical guide for applications of gene expression profiling in human health risk assessment of environmental chemicals. Regul. Toxicol. Pharmacol. 72 (2), 292–309. 10.1016/j.yrtph.2015.04.010 25944780 PMC7970737

[B7] Carvalhais L. C. Dennis P. G. Tyson G. W. Schenk P. M. (2012). Application of metatranscriptomics to soil environments. J. Microbiol. methods 91 (2), 246–251. 10.1016/j.mimet.2012.08.011 22963791

[B8] Chang Y. Rager J. E. Tilton S. C. (2021). Linking coregulated gene modules with polycyclic aromatic hydrocarbon-related cancer risk in the 3D human bronchial epithelium. Chem. Res. Toxicol. 34 (6), 1445–1455. 10.1021/acs.chemrestox.0c00333 34048650 PMC8560124

[B9] Conesa A. Madrigal P. Tarazona S. Gomez-Cabrero D. Cervera A. McPherson A. (2016). A survey of best practices for RNA-seq data analysis. Genome Biol. 17 (1), 13–19. 10.1186/s13059-016-0881-8 26813401 PMC4728800

[B10] Dasgupta S. Leong C. Simonich M. T. Truong L. Liu H. Tanguay R. L. (2022). Transcriptomic and long-term behavioral deficits associated with developmental 3.5 GHz radiofrequency radiation exposures in zebrafish. Environ. Sci. Technol. Lett. 9 (4), 327–332. 10.1021/acs.estlett.2c00037 35434172 PMC9009179

[B11] De Toma I. Sierra C. Dierssen M. (2021). Meta-analysis of transcriptomic data reveals clusters of consistently deregulated gene and disease ontologies in Down syndrome. PLoS Comput. Biol. 17 (9), e1009317. 10.1371/journal.pcbi.1009317 34570756 PMC8496798

[B12] Dean J. L. Zhao Q. J. Lambert J. C. Hawkins B. S. Thomas R. S. Wesselkamper S. C. (2017). Editor's highlight: Application of gene set enrichment analysis for identification of chemically induced, biologically relevant transcriptomic networks and potential utilization in human health risk assessment. Toxicol. Sci. 157 (1), 85–99. 10.1093/toxsci/kfx021 28123101 PMC6106787

[B13] Dobin A. Davis C. A. Schlesinger F. Drenkow J. Zaleski C. Jha S. (2013). Star: Ultrafast universal RNA-seq aligner. Bioinformatics 29 (1), 15–21. 10.1093/bioinformatics/bts635 23104886 PMC3530905

[B14] Fasold M. Binder H. (2012). Estimating RNA-quality using GeneChip microarrays. BMC genomics 13 (1), 186–225. 10.1186/1471-2164-13-186 22583818 PMC3519671

[B15] Gao C. Weisman D. Lan J. Gou N. Gu A. Z. (2015). Toxicity mechanisms identification via gene set enrichment analysis of time-series toxicogenomics data: Impact of time and concentration. Environ. Sci. Technol. 49 (7), 4618–4626. 10.1021/es505199f 25785649 PMC6321746

[B16] Hu W. Yang S. Shimada Y. Münch M. Marín-Juez R. Meijer A. H. (2019). Infection and RNA-seq analysis of a zebrafish tlr2 mutant shows a broad function of this toll-like receptor in transcriptional and metabolic control and defense to Mycobacterium marinum infection. BMC genomics 20 (1), 878–918. 10.1186/s12864-019-6265-1 31747871 PMC6869251

[B17] Huang W. Zheng S. Wang X. Cai Z. Xiao J. Liu C. (2020). A transcriptomics-based analysis of toxicity mechanisms of zebrafish embryos and larvae following parental Bisphenol A exposure. Ecotoxicol. Environ. Saf. 205, 111165. 10.1016/j.ecoenv.2020.111165 32836160

[B18] Janjic A. Wange L. E. Bagnoli J. W. Geuder J. Nguyen P. Richter D. (2022). Prime-seq, efficient and powerful bulk RNA sequencing. Genome Biol. 23 (1), 88–27. 10.1186/s13059-022-02660-8 35361256 PMC8969310

[B19] Jarvis S. Birsa N. Secrier M. Fratta P. Plagnol V. (2020). A comparison of low read depth QuantSeq 3′ sequencing to Total RNA-Seq in FUS mutant mice. Front. Genet. 11, 562445. 10.3389/fgene.2020.562445 33329699 PMC7717943

[B20] Jiang M. Xiao Y. Weigao E. Ma L. Wang J. Chen H. (2021). Characterization of the zebrafish cell landscape at single-cell resolution. Front. Cell Dev. Biol. 9, 743421. 10.3389/fcell.2021.743421 34660600 PMC8517238

[B21] Joseph P. (2017). Transcriptomics in toxicology. Food Chem. Toxicol. 109 (Pt 1), 650–662. 10.1016/j.fct.2017.07.031 28720289 PMC6419952

[B22] Kamitani M. Kashima M. Tezuka A. Nagano A. J. (2019). Lasy-seq: A high-throughput library preparation method for RNA-seq and its application in the analysis of plant responses to fluctuating temperatures. Sci. Rep. 9 (1), 7091–7114. 10.1038/s41598-019-43600-0 31068632 PMC6506593

[B23] Katz Y. Wang E. T. Airoldi E. M. Burge C. B. (2010). Analysis and design of RNA sequencing experiments for identifying isoform regulation. Nat. methods 7 (12), 1009–1015. 10.1038/nmeth.1528 21057496 PMC3037023

[B24] Kelkar D. S. Provost E. Chaerkady R. Muthusamy B. Manda S. S. Subbannayya T. (2014). Annotation of the zebrafish genome through an integrated transcriptomic and proteomic analysis. Mol. Cell. proteomics 13 (11), 3184–3198. 10.1074/mcp.m114.038299 25060758 PMC4223501

[B25] Kimmel C. B. Ballard W. W. Kimmel S. R. Ullmann B. Schilling T. F. (1995). Stages of embryonic development of the zebrafish. Dev. Dyn. 203 (3), 253–310. 10.1002/aja.1002030302 8589427

[B26] Korotkevich G. Sukhov V. Budin N. Shpak B. Artyomov M. N. Sergushichev A. (2016). Fast gene set enrichment analysis. BioRxiv, 060012.

[B27] Łabaj P. P. Leparc G. G. Linggi B. E. Markillie L. M. Wiley H. S. Kreil D. P. (2011). Characterization and improvement of RNA-Seq precision in quantitative transcript expression profiling. Bioinformatics 27 (13), i383–i391. 10.1093/bioinformatics/btr247 21685096 PMC3117338

[B28] Landry J. J. Pyl P. T. Rausch T. Zichner T. Tekkedil M. M. Stutz A. M. (2013). The genomic and transcriptomic landscape of a HeLa cell line. G3 (Bethesda) 3 (8), 1213–1224. 10.1534/g3.113.005777 23550136 PMC3737162

[B29] Langmead B. Salzberg S. L. (2012). Fast gapped-read alignment with Bowtie 2. Nat. methods 9 (4), 357–359. 10.1038/nmeth.1923 22388286 PMC3322381

[B30] Lawson N. D. Li R. Shin M. Grosse A. Yukselen O. Stone O. A. (2020). An improved zebrafish transcriptome annotation for sensitive and comprehensive detection of cell type-specific genes. Elife 9, e55792. 10.7554/elife.55792 32831172 PMC7486121

[B31] Li Y. Yang H. Zhang H. Liu Y. Shang H. Zhao H. (2020). Decode-seq: A practical approach to improve differential gene expression analysis. Genome Biol. 21 (1), 66–67. 10.1186/s13059-020-01966-9 32200760 PMC7087377

[B32] Love M. I. Huber W. Anders S. (2014). Moderated estimation of fold change and dispersion for RNA-seq data with DESeq2. Genome Biol. 15 (12), 550–621. 10.1186/s13059-014-0550-8 25516281 PMC4302049

[B33] Ma F. Fuqua B. K. Hasin Y. Yukhtman C. Vulpe C. D. Lusis A. J. (2019). A comparison between whole transcript and 3’RNA sequencing methods using Kapa and Lexogen library preparation methods. BMC genomics 20 (1), 9–12. 10.1186/s12864-018-5393-3 30616562 PMC6323698

[B34] Mandrell D. Truong L. Jephson C. Sarker M. R. Moore A. Lang C. (2012). Automated zebrafish chorion removal and single embryo placement: Optimizing throughput of zebrafish developmental toxicity screens. J. laboratory automation 17 (1), 66–74. 10.1177/2211068211432197 PMC3327291 22357610

[B35] Metikala S. Casie Chetty S. Sumanas S. (2021). Single-cell transcriptome analysis of the zebrafish embryonic trunk. Plos one 16 (7), e0254024. 10.1371/journal.pone.0254024 34234366 PMC8263256

[B36] Moll P. Ante M. Seitz A. Reda T. (2014). QuantSeq 3′ mRNA sequencing for RNA quantification. Nat. methods 11 (12), i–iii. 10.1038/nmeth.f.376

[B37] Nichols R. G. Zhang J. Cai J. Murray I. A. Koo I. Smith P. B. (2019). Metatranscriptomic analysis of the mouse gut microbiome response to the persistent organic pollutant 2, 3, 7, 8-tetrachlorodibenzofuran. Metabolites 10 (1), 1. 10.3390/metabo10010001 31861317 PMC7022680

[B38] Pan K-H. Lih C-J. Cohen S. N. (2005). Effects of threshold choice on biological conclusions reached during analysis of gene expression by DNA microarrays. Proc. Natl. Acad. Sci. 102 (25), 8961–8965. 10.1073/pnas.0502674102 15951424 PMC1149502

[B39] Raudvere U. Kolberg L. Kuzmin I. Arak T. Adler P. Peterson H. (2019). g:Profiler: a web server for functional enrichment analysis and conversions of gene lists (2019 update). Nucleic acids Res. 47 (W1), W191–W198. 10.1093/nar/gkz369 31066453 PMC6602461

[B40] Rericha Y. Cao D. Truong L. Simonich M. T. Field J. A. Tanguay R. L. (2022). Sulfonamide functional head on short-chain perfluorinated substance drives developmental toxicity. Iscience 25 (2), 103789. 10.1016/j.isci.2022.103789 35146398 PMC8819378

[B41] Shankar P. Geier M. C. Truong L. McClure R. S. Pande P. Waters K. M. (2019). Coupling genome-wide transcriptomics and developmental toxicity profiles in zebrafish to characterize polycyclic aromatic hydrocarbon (PAH) hazard. Int. J. Mol. Sci. 20 (10), 2570. 10.3390/ijms20102570 31130617 PMC6566387

[B42] Shankar P. McClure R. S. Waters K. M. Tanguay R. L. (2021). Gene co-expression network analysis in zebrafish reveals chemical class specific modules. BMC genomics 22 (1), 658–720. 10.1186/s12864-021-07940-4 34517816 PMC8438978

[B43] Sigurgeirsson B. Emanuelsson O. Lundeberg J. (2014). Sequencing degraded RNA addressed by 3'tag counting. PloS one 9 (3), e91851. 10.1371/journal.pone.0091851 24632678 PMC3954844

[B44] Simillion C. Liechti R. Lischer H. E. Ioannidis V. Bruggmann R. (2017). Avoiding the pitfalls of gene set enrichment analysis with SetRank. BMC Bioinforma. 18 (1), 151–214. 10.1186/s12859-017-1571-6 PMC5336655 28259142

[B45] Tandonnet S. Torres T. T. (2017). Traditional versus 3′ RNA-seq in a non-model species. Genomics data 11, 9–16. 10.1016/j.gdata.2016.11.002 27909684 PMC5124356

[B46] Tatarakis D. Cang Z. Wu X. Sharma P. P. Karikomi M. MacLean A. L. (2021). Single-cell transcriptomic analysis of zebrafish cranial neural crest reveals spatiotemporal regulation of lineage decisions during development. Cell Rep. 37 (12), 110140. 10.1016/j.celrep.2021.110140 34936864 PMC8741273

[B47] Torres T. T. Metta M. Ottenwälder B. Schlötterer C. (2008). Gene expression profiling by massively parallel sequencing. Genome Res. 18 (1), 172–177. 10.1101/gr.6984908 18032722 PMC2134766

[B48] Tveit A. T. Urich T. Svenning M. M. (2014). Metatranscriptomic analysis of arctic peat soil microbiota. Appl. Environ. Microbiol. 80 (18), 5761–5772. 10.1128/aem.01030-14 25015892 PMC4178616

[B49] Wang Z. Gerstein M. Snyder M. (2009). RNA-seq: A revolutionary tool for transcriptomics. Nat. Rev. Genet. 10 (1), 57–63. 10.1038/nrg2484 19015660 PMC2949280

[B50] Westerfield M. (2007). THE ZEBRAFISH BOOK. 5th Edn. A guide for the laboratory use of zebrafish (Danio rerio). Eugene: University of Oregon Press. Paperback.

[B51] Zayed Y. Qi X. Peng C. (2019). Identification of novel MicroRNAs and characterization of MicroRNA expression profiles in zebrafish ovarian follicular cells. Front. Endocrinol. 10, 518. 10.3389/fendo.2019.00518 PMC6684945 31417497

[B52] Zheng M. Lu J. Zhao D. (2018). Toxicity and transcriptome sequencing (RNA-seq) analyses of adult zebrafish in response to exposure carboxymethyl cellulose stabilized iron sulfide nanoparticles. Sci. Rep. 8 (1), 8083–8111. 10.1038/s41598-018-26499-x 29795396 PMC5967324

