# Supplementary figures and images for: 3′ RNA-seq is superior to standard RNA-seq in cases of sparse data but inferior at identifying toxicity pathways in a model organism
Source: Front Bioinform. 2023 Jul 27;3:1234218. doi: 10.3389/fbinf.2023.1234218 (PMC10414111; doi:10.3389/fbinf.2023.1234218)

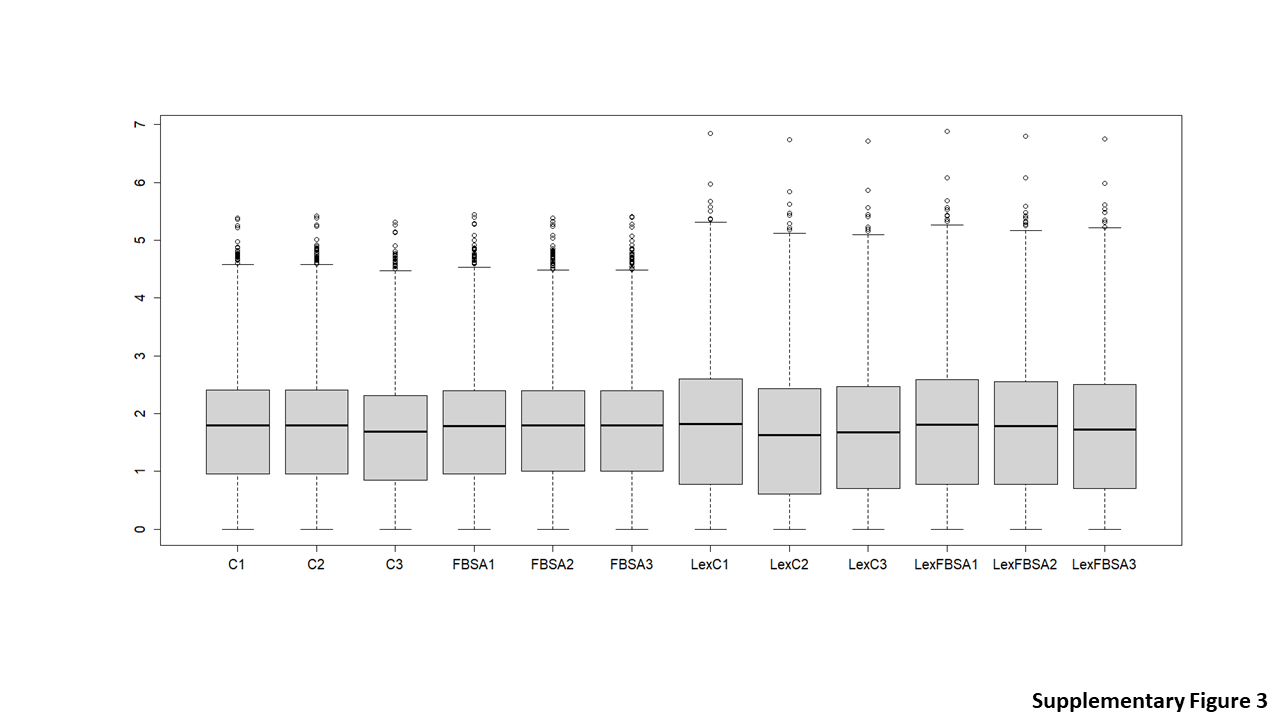

Supplement: Supplementary file 1 [file Image3.tif]

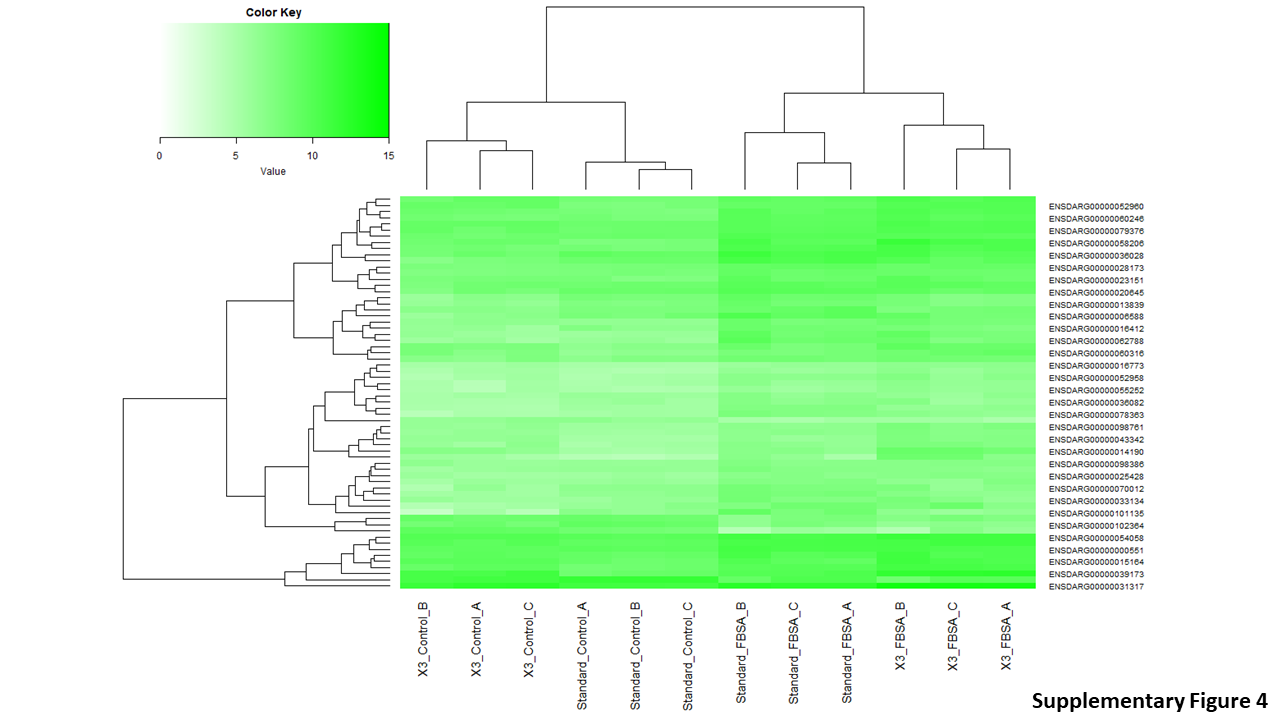

Supplement: Supplementary file 2 [file Image4.tif]

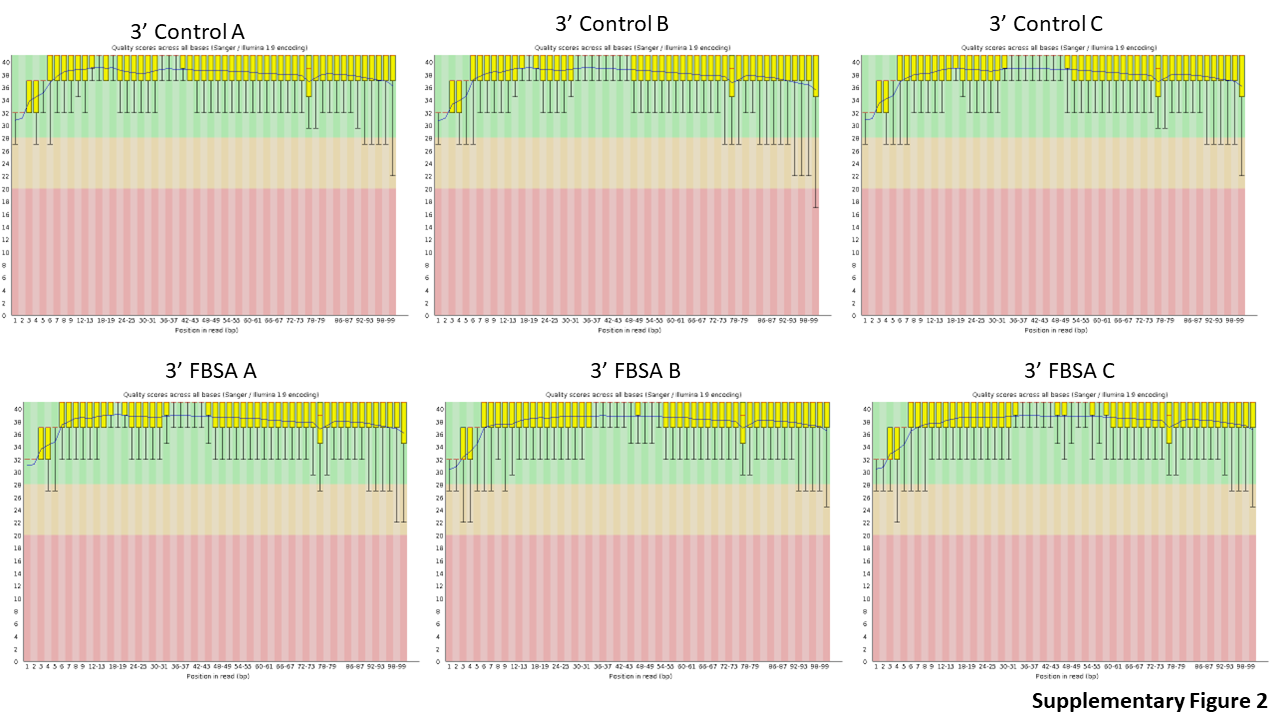

Supplement: Supplementary file 3 [file Image2.tif]

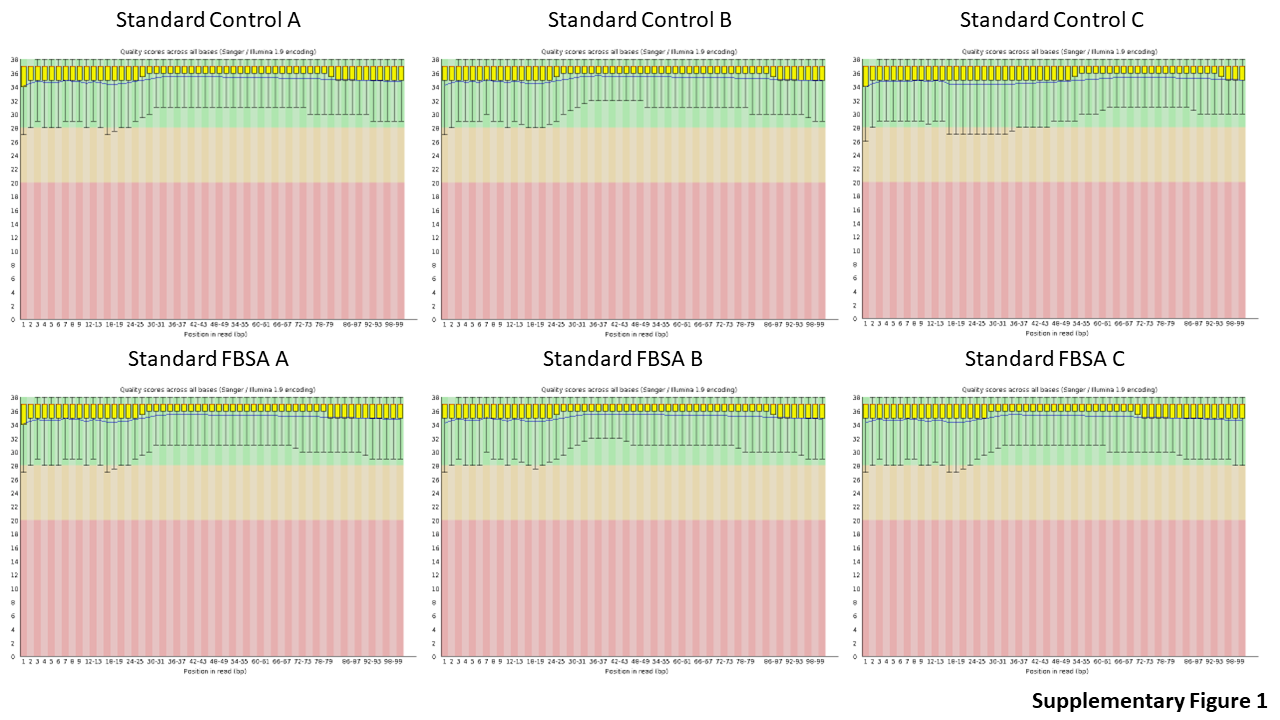

Supplement: Supplementary file 4 [file Image1.tif]

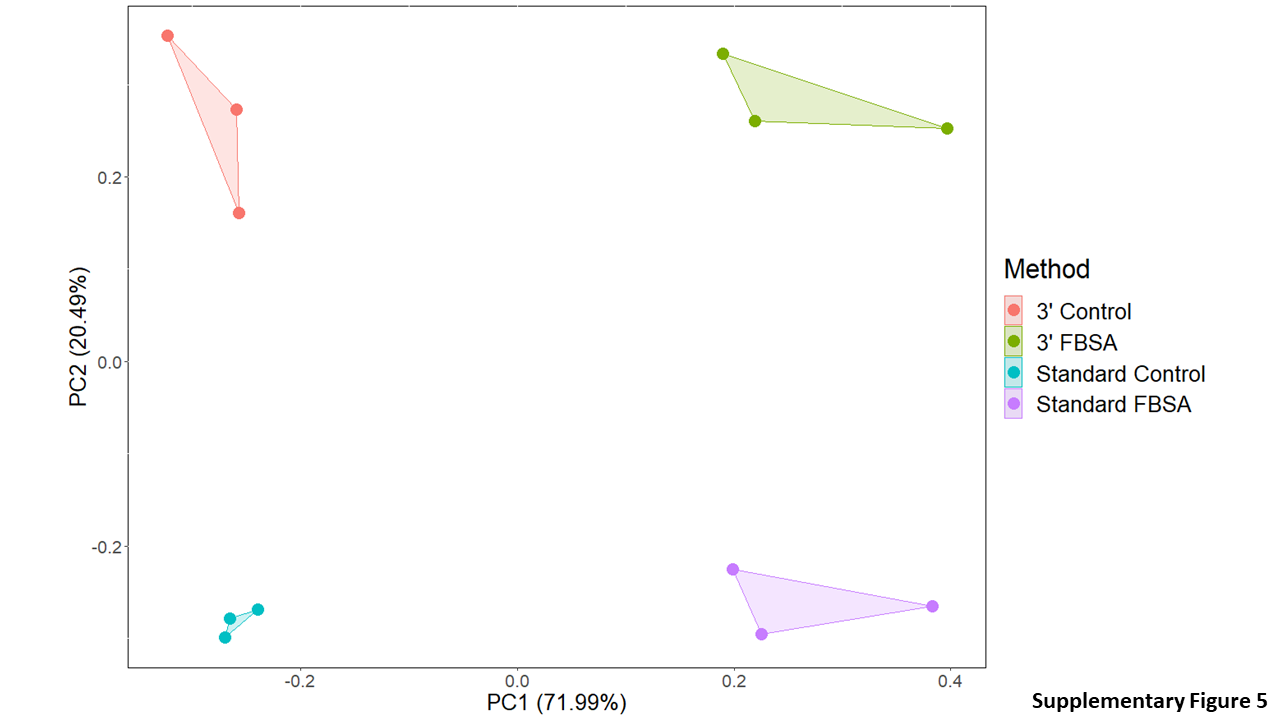

Supplement: Supplementary file 6 [file Image5.tif]
